# Proteomic and Glycomic Characterization of Rice Chalky Grains Produced Under Moderate and High-temperature Conditions in Field System

**DOI:** 10.1186/s12284-016-0100-y

**Published:** 2016-05-31

**Authors:** Kentaro Kaneko, Maiko Sasaki, Nanako Kuribayashi, Hiromu Suzuki, Yukiko Sasuga, Takeshi Shiraya, Takuya Inomata, Kimiko Itoh, Marouane Baslam, Toshiaki Mitsui

**Affiliations:** Graduate School of Science and Technology, Niigata University, Niigata, 950-2181 Japan; Department of Applied Biological Chemistry, Niigata University, Niigata, 950-218 Japan; Present address: Niigata Crop Research Center, Niigata Agricultural Research Institute, Nagaoka, 940-0826 Japan

**Keywords:** Amylase, Chaperones, Endosperm, Environment, Heat shock protein, High-temperature stress, Late embryogenesis abundant (LEA) protein, Grain chalkiness, Starch granule, Stress-related protein

## Abstract

**Background:**

Global climate models predict an increase in global mean temperature and a higher frequency of intense heat spikes during this century. Cereals such as rice (*Oryza sativa* L.) are more susceptible to heat stress, mainly during the gametogenesis and flowering stages. During periods of high temperatures, grain filling often causes serious damage to the grain quality of rice and, therefore, yield losses. While the genes encoding enzymes involved in carbohydrate metabolism of chalky grains have been established, a significant knowledge gap exists in the proteomic and glycomic responses to warm temperatures in situ. Here, we studied the translucent and opaque characters of high temperature stressed chalky grains of 2009 and 2010 (ripening temperatures: 24.4 and 28.0 °C, respectively).

**Results:**

Appearance of chalky grains of both years showed some resemblance, and the high-temperature stress of 2010 remarkably extended the chalking of grain. Scanning electron microscopic observation showed that round-shaped starch granules with numerous small pits were loosely packed in the opaque part of the chalky grains. Proteomic analyzes of rice chalky grains revealed deregulations in the expression of multiple proteins implicated in diverse metabolic and physiological functions, such as protein synthesis, redox homeostasis, lipid metabolism, and starch biosynthesis and degradation. The glycomic profiling has shown slight differences in chain-length distributions of starches in the grains of 2009-to-2010. However, no significant changes were observed in the chain-length distributions between the translucent and opaque parts of perfect and chalky grains in both years. The glucose and soluble starch contents in opaque parts were increased by the high-temperature stress of 2010, though those in perfect grains were not different regardless of the environmental changes of 2009-to-2010.

**Conclusion:**

Together with previous findings on the increased expression of α-amylases in the endosperm, these results suggested that unusual starch degradation rather than starch synthesis is involved in occurring of chalky grains of rice under the high-temperature stress during grain filling period.

**Electronic supplementary material:**

The online version of this article (doi:10.1186/s12284-016-0100-y) contains supplementary material, which is available to authorized users.

## Background

Global climate change is one of the most serious environmental threats we face today. Since the early 20th century, the average surface temperature of the Earth has unusually increased by about 0.8 °C coupled with the rapid warming of 0.6 °C over the past three decades. Climate change is also projected to have significant impacts on crop production (IPCC 2013). In 80 % of the rice harvested areas in Japan climate variability was more important and the explanation is on account of temperature variability (Ray et al. 2015). For every 1 °C increase in temperature, there was a 6.6 % decrease in yield from the current values for early rice, 5.2 % for late rice, and 8.2 % for single-cropped rice (Defeng and Shaokai 1995). The abnormal high temperature during rice endosperm development and grain filling periods can change the chemical ingredients of rice caryopses such as starch and storage proteins and the contents of fatty acid, thus causing a decrease in grain yield, quality and, hence, price. The occurrence of chalky grains of rice is increased by high-temperature stress during grain filling (Nagato and Ebata 1965). Morita et al. (2016) showed that chalky grains of *japonica* cultivars have been produced under temperatures more than 26 °C during the grain-filling period. Daily mean air temperatures of 26 °C during grain filling are becoming frequent in Japan (Usui et al. 2014). Many research groups have studied morphological characteristics of chalky grains, showing that abnormal starch granules were loosely packed in the chalky grains (Evers and Juliano 1976; Tashiro and Wardlaw 1991; Kim et al. 2000; Lisle et al. 2000; Singh et al. 2003; Ishimaru et al. 2009). Kernels with chalky have a lower density of starch granules than do vitreous ones and are more prone to breakage during milling. The surface of round-shaped starch granules in chalky grains caused by high-temperature stress had small pits occasionally (Tashiro and Wardlaw 1991). It should be noted that the feature of change of granule surface was similar to that occurring during germination (Fuwa et al. 1977). The environmental temperature at the grain filling stage has been reported to influence the starch composition in rice grains (Asaoka et al. 1984; Inouchi et al. 2000; Lisle et al. 2000; Umemoto and Terashima 2002; Cheng et al. 2005; Yamakawa et al. 2007; Mitsui et al. 2016). High temperature caused a reduction in the amylose contents and changed the fine structure of amylopectin (Asaoka et al. 1984; Inouchi et al. 2000; Cheng et al. 2005), suggesting that the unusual expression of the starch synthesizing enzymes is a possible key factor causing the chalky grains of rice (Nishi et al. 2001; Tanaka et al. 2004). On the other hand, surface of the starch granules showed clear `erosion´ with multiple small pits suggesting an attack by α-amylases (Zakaria et al. 2002; Iwasawa et al. 2009); the suppression of α-amylase genes by RNA interference improved the appearance quality of rice grains ripened under heat stress (Hakata et al. 2012). However, the mechanism of grain chalkiness under high-temperature stress is considerably complicated and still remains poorly understood: a disorder of photosynthesis, translocation efficiency, source-sink relationship, and protein expression in ripening seeds may involve in such chalking mechanism. Many quantitative trait loci (QTLs) controlling grain appearance quality have been identified in populations derived from crosses between *japonica* cultivars (Tabata et al. 2007; Ebitani et al. 2008; Kobayashi et al. 2013; Ishimaru et al. 2016), between *japonica* and *indica* cultivars (He et al. 1999; Wan et al. 2005; Ishimaru et al. 2016), *indica* cultivars (Mei et al. 2013), and between *O. sativa* and *O. glaberrima* (Li et al. 2004). Therefore, understanding of the mechanisms of grain chalking is indispensable to develop a strategy for reducing the high rate of chalky grains under the likely scenario of global warming (Lin et al. 2014).

On the other hand, in addition to starch, proteins account for 6–10 % of the dry mass and are important for the nutrition, cooking, and brewing quality of rice grains (Bressani et al. 1971; Hamaker 1994). Therefore, the importance of rice endosperm proteins should not be underestimated. The lack of perfect correlation of mRNA and protein levels during heat treatment has pushed the researchers to explore insights into the proteomic basis, since this technique provides a more direct assessment of the actual proteins performing the signaling, enzymatic, regulatory and structural functions encoded by the genome and transcriptome (Echevarría-Zomeño et al. 2015). Considerable work on rice grains proteomics has been carried out using two-dimensional polyacrylamide gel electrophoresis (2D-PAGE) and gel-free-based shotgun procedures (Koller et al. 2002; Lin et al. 2005, 2014; Xu et al. 2008; Lee and Koh 2011). Non-redundant proteins of 4,172 with a wide range of molecular weight (5.2–611 kDa) and pI values (pH 2.9–12.6) in developing and mature grains of rice has been identified by using a label-free shotgun technique (Lee and Koh 2011). The ontology categories of 52 including the carbohydrate metabolic process, transport, localization, lipid metabolic process, and secondary metabolic process were enriched. Expression analyzes of functionally categorized protein groups showed dynamic changes of metabolisms during rice grain development. As a noteworthy observation, proteins involved in glycolysis, citric acid cycle, lipid metabolism, and proteolysis accumulated at higher levels in mature grain than those of developing stages (Lee and Koh 2011). This probably indicates that the preparation of materials required in germination occurred until the seeds were fully matured and dried.

Proteomic information of rice grains in the anthesis, ripening, and maturing stages under heat stress has been gradually accumulated. The anthesis and early ripening stages are known to be highly sensitive to heat stress. Gel-based proteomic analyzes of different genotype anthers prepared from rice plants treated with high (38 °C) and control (29 °C) temperature at anthesis were carried out (Jagadish et al. 2008). Both cold (19 kDa) and heat (24 kDa) shock proteins were significantly up-regulated in a heat-tolerant genotype N22, these possibly contributing to the greater heat tolerance of N22. Heat stress (35/30 °C day/night) during an early stage of caryopsis development reduced the expression of starch granule-bound starch synthase (Wx) and prolamin, but enhanced the expression of dnaK-type hsp70 and glutelins in comparison with those in control temperature (30/25 °C) (Lin et al. 2005). In addition, heat stress response of several different cultivars including high-chalky types were analyzed, the results showing that sHSP was positively correlated with the appearance of chalky kernels (Lin et al. 2005). In recent studies, accumulation of all classes of storage proteins was increased at early filling stage under heat stress (35/30 °C), whereas the prolamin accumulation was decreased at maturation and desiccation stages (Lin et al. 2010). On the other hand, Lin et al. (2014) showed that up-regulation of proteins involved in starch accumulation and down-regulation of ER proteins, PDIL 2–3 and BiP, were observed in the chalky tissue of notched-belly mutant appeared regardless of the environmental stress, employing a comparative proteomic analysis by iTRAQ (isobaric tags for relative and absolute quantification). Understanding protein expression patterns and its respective posttranslational modifications in chalky grain is of fundamental importance for targeting rice quality under temperatures variability.

In the present investigation, we characterized chalky and perfect grains of rice harvested in Niigata, central Japan, of 2009 and 2010 employing proteomic and glycomic techniques to provide a better understanding of the mechanisms of chalky formation under hot season in field conditions. 2010 was the hottest year since Japan began keeping records; on the other hand, 2009 was an average crop year. Our results provide a comprehensive view of proteome and glycomic characterization under temperature variability. We consider which proteins, possibly, involved in the grain chalking under the paddy field conditions.

## Results and discussion

### Rice grains under high temperature showed chalky appearance

Perfect and chalky grains of rice (cv. *Koshihikari*) were harvested at a paddy field of Sanjo city (Niigata, Japan) in 2009 and 2010. The average temperature of heading and ripening period of Koshihikari in 2010 (28.0 °C) was much higher than that in 2009 (24.4 °C). The 1,000-kernel weight of chalky grains in 2010 (15.61 g) was apparently small compared with the 2009 chalky grains (16.97 g), but a variation of the 1,000-kernel weight of the perfect grains in 2009 and 2010 (21.62 and 21.99 g, respectively) was indistinguishable. The volumes (length x width x thickness) of 2009 perfect, 2010 perfect, 2009 chalky, and 2010 chalky grains were 29.15, 28.73, 23.85 and 23.84 mm^3^, respectively. Thus, the size of the chalky grain was smaller than that of the perfect grain, whereas there was no difference between the sizes in 2009 and 2010 chalky grains. The decrease in rice grain length and width might be related with the reduction in average endosperm cell area observed under high temperature (Morita et al. 2005). Consequently, it has been suggested that the flow of C and N to the grain (Mohammadi et al. 2010), and insufficient supply of photosynthates from source to sink organ and carbohydrate deficit (Liu et al. 2009; Kanno and Makino 2010; Shi et al. 2013) caused by increased temperature could be causes of chalkiness.

**Fig. 1:**
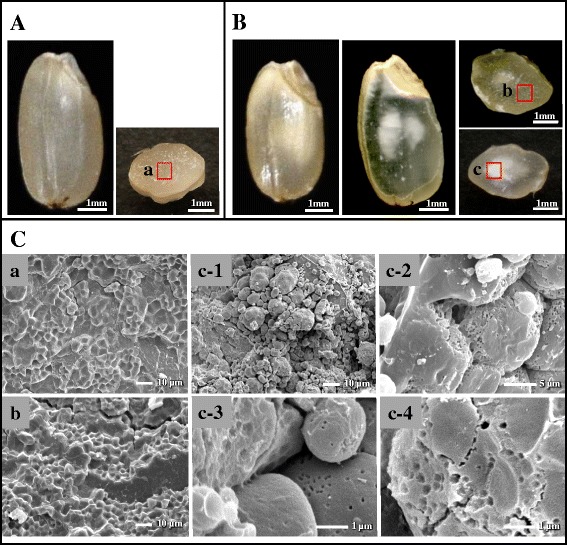
Morphological characteristics of chalky grain of rice. (A) Perfect grain. Left and right panels show pictures of whole grain and horizontal slice, respectively. (B) Chalky grain. Left, whole grain; middle, vertical slice; right, horizontal slices. (C) SEM pictures. Center part of perfect grain (a), and translucent (b) and opaque (c) parts of chalky grain were subjected to SEM observation. Magnifications were x1,000 (a,b,c-1), x4,000 (c-2) and x10,000 (c-3, c-4), respectively

The ratios of the opaque region to the whole area in 2009 and 2010 chalky grains were estimated to be 61.1 ± 10.2 and 78.4 ± 13.6 % (*n* = 50), respectively. These results strongly suggest that the high-temperature stress further extended the chalking of grain. Starch granule morphologies of the perfect and chalky grains were analyzed by scanning electron microscopy (SEM). In the perfect grain (Fig. 1a), the starch granules were tightly packed in the endosperm cells, and the shape of starch granule was polygonal with sharp edges (Fig. 1Ca). In the chalky grain (Fig. 1b), the starch granules in the translucent part of chalky grains had similar tight packing and shape (Fig. 1Cb) compared to the perfect grain. While in the opaque part, the starch granules were loosely packed, and some granules had a round shape with several small pits (Fig. 1c; c-1, 2, 3 and 4). The numerous pits were frequently observed upon the surface of round-shaped starch granules in the chalky endosperm caused by high-temperature stress of grain filling period (Evers and Juliano 1976, Tashiro and Wardlaw 1991). It is noteworthy that the present result of SEMs observation confirmed display signs of pitting on the starch granules in the chalky grains (Fig. 1c; c-1, 2, 3 and 4). The chalky zone sometimes occurs midway between the center and peripheral part of the endosperm in the developing kernels under high-temperature conditions. The ring-shaped chalkiness could be a cell-specific event associated with the disruption of starch accumulation. Similar observations made for cell layers in rice endosperm showed that inadequate starch accumulation occurs from the center towards outward in the endosperms (Wada et al. 2014). The increase of the frequency of pitting in starch granules with high temperature is presumably due to the premature autolysis of starch. This is similar to the premature autolysis reported in the case of wheat and maize kernels subjected to high temperatures (Tashiro and Wardlaw 1990, 1991; Commuri and Jones 1999).

For analyzing changes in the proteome and glycome of grains, the translucent and opaque parts of chalky grains and the central parts of perfect grains were prepared as follows: central part of perfect grain in 2009 (PG 2009), translucent part of chalky grain in 2009 (tCG 2009), opaque part of chalky grain in 2009 (oCG 2009), central part of perfect grain in 2010 (PG 2010), translucent part of chalky grain in 2010 (tCG 2010), and opaque part of chalky grain in 2010 (oCG 2010).

### Proteomic profile of chalky grains caused under different temperature conditions

**Fig. 2:**
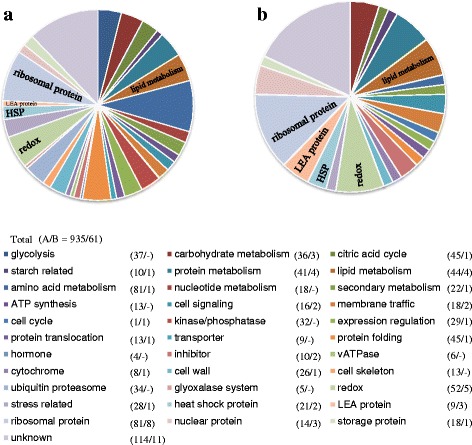
Proteome of perfect and chalky grains of rice. Center part of perfect grains and opaque part of chalky grains harvested in 2009 and 2010 were subjected to protein extraction, followed by in solution shotgun proteomic analysis with iTRAQ labeling. (A/B): **a**, Total identified proteins (935); **b** ≧ Two-fold up- or down-regulated proteins in the chalky grains of 2010 (61)

To characterize the proteome involved in the mechanism of grain chalking, we carried out a quantitative shotgun proteomic analysis of starchy endosperms prepared from the opaque part of chalky grains (oCG 2009 and oCG2010) and the corresponding part of perfect grains harvested in 2009 and 2010 (PG 2009 and PG2010). The extracted proteins were trypsin-digested and labeled by iTRAQ (isobaric tag for relative and absolute quantitation), followed by tandem mass spectrometry (MS/MS) analysis. Analysis of protein extracts from the different samples resulted in the detection 938 of roteins (Fig. 2a, Additional file 1: Table S1). This analysis revealed that the expression of 61 proteins, 6.5 % of all identified proteins, were deregulated (more than 2.0-fold difference relative to PG control; P value < 0.05) in the oCG 2010 (Fig. 2b, Additional file 1: Table S1). Among this population, 41 genes were up-regulated and 20 genes were down-regulated.

**Fig. 3:**
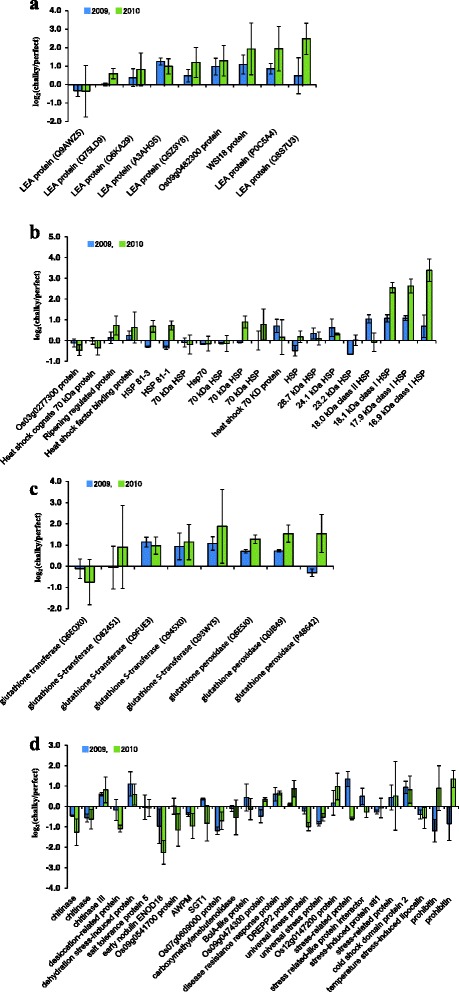
Proteome of stress-related proteins in chalky grains. Center part of perfect grains and opaque part of chalky grains harvested in 2009 and 2010 were subjected to protein extraction, followed by in solution shotgun proteomic analysis with iTRAQ labeling. **a** Late embryogenesis abundant proteins; **b** Heat shock proteins; **c** Glutathione redox regulation; **d** other stress-related proteins. Proteins were categorized by NCBI databases. Values are represented as mean ± s.d. (*n* = 3)

To determine the biological processes affected by high temperature, an analysis of proteins using the InterPro-scan was carried out. This research revealed that chalkiness clearly leads to the differential expression of proteins and suggest that the energy and metabolic pathways are highly disrupted; other categories of proteins may involve in protecting the cellular damage under high-temperature stress (Fig. 2). The annotation of protein sequences using InterProScan permitted to fall them into 33 categories. In the molecular functional group, the identified proteins; ribosomal, redox, LEA and HSP were ranked at the top of the category occupancy, suggesting that the relevant functions were important in the response to high-temperature stimuli. It is thus conceivable that, as illustrated in Fig. 2b, differential expression of these proteins are partially the consequence of the apparition of chalky tissue. Under high-temperature stress, reactive oxygen species are generated, and cell membrane integrity is often lost leading to cell death. The heat-stressed plants signal for reprogramming cellular metabolism, either by increasing or decreasing the transcriptional and translational events. As shown in Fig. 3, we classified the detected stress-related proteins involved in oCG during 2009 and 2010 into four major groups according to their physiological functions: late embryogenesis abundant (LEA) (Fig. 3a), heat shock protein (HSP) (Fig. 3b), glutathione redox regulation (Fig. 3c) and other stress-related proteins (Fig. 3d).

The results suggested that in both oCG of 2009 and 2010 LEA proteins were markedly accumulated, being more abundant in oCG 2010 hot season (+ ~ 4 °C than 2009). In oCG 2010, Q75LD9, Q8S7U3, like other six identified LEA proteins, increased in abundance, while Q9AWZ5 slightly decreased in abundance The majority of LEA proteins display a preponderance of hydrophilic and charged amino acid residues (Xiao et al. 2007). Also, considerable evidence suggests that LEA proteins are involved in desiccation resistance (Ingram and Bartels 1996), a variety of mechanisms for achieving this end have been proposed including protecting cellular structures from the effects of water loss by retention of water, sequestration of ions, direct protection of other proteins or membranes, or renaturation of unfolded proteins (Cuming 1999; Bray et al. 2000; Olvera-Carrillo et al. 2011).

HSPs can be used to stabilize protein conformation, prevent aggregation, and, therefore, maintain non-native proteins in a competent state for subsequent refolding in plants under heat stress (Morimoto 2002; Wang et al. 2004; Zi et al. 2013). The significant up-regulation of the HSPs is a key part of the heat shock response and is induced primarily by heat shock factors (HSFs) such as temperatures variability, drought, salinity, cold and chemicals (Scarpeci et al. 2008; Al-Whaibi 2011; Liao et al. 2013). Among the HSPs illustrated in this study, the amount of the HSP 81–1, HSP 81–3, and 70 kDa HSP involved strong up-regulated in oCG 2010 -hot season-, while they were down-regulated in oCG 2009 -moderate temperature season-. The HSP70 family, group of molecular chaperones, such as BiP1 and BiP2 maintains polypeptides in an unfolded state (Lin et al. 2014). There is evidence that changes in BiP protein levels induce endoplasmic reticulum stress, and BiP overexpression affects the accumulation of seed storage proteins and, then, results in an opaque phenotype in the whole endosperm of rice (Yasuda et al. 2009). In addition, small heat-shock protein (sHSPs) encoding genes (HSP16.9A, HSP17.9A, HSP18.1) were up-regulated in oCG 2010, although the oCG 2009 moderately accumulated these sHSP (Fig. 3b). Among the various plant Hsps (i.e. HSP100, HSP90, HSP70, and HSP20), sHSPs have been identified to expressed in maximal amounts under high-temperature stress. They are also up-regulated in rice caryopses during the grain milky stage (Lin et al. 2005; Liao et al. 2013). Our data were supported by a number of previous studies. HSP70 was reported to potentially be involved in a repair function after desiccation rather than biochemical stabilization in the dry state for *Richtersius coronifer* (Zi et al. 2013), whereas sHSPs associate with nuclei, cytoskeleton, and membranes, and as molecular chaperones they bind partially denatured proteins, thereby preventing irreversible protein aggregation during stress. sHSPs were positively correlated with the appearance of chalky kernels (Lin et al. 2005). In corroboration with another report (Das et al. 2015), the present results support the view that the induction of sHSPs suggests their function in re-establishing normal protein confirmation and thus cellular homeostasis. Besides, this study revealed that the high temperature in 2010 (+ ~ 4 °C) increased the abundance of high molecular weight HSPs. The HSP81.3 and 81.1, of the HSP90 gene family, are associated with different polypeptides serving a general mode of cellular activities. Moreover, the expression of the HSP90 genes and mRNA accumulation in plants and calli were strongly induced by high temperature (Milioni and Hatzopoulos 1997). HSP data indicate that the HSP90 (HSP81-1, HSP81-3) and 70 kDa HSP may play an important role at the chalky tissue formation. It is believed that this diversification of HSP reflects an adaptation to tolerate the heat stress in chalky rice grains. Moreover, the physiological role of chaperones like HSP90 in plants remains poorly understood. Needless to say, further research will be necessary to study the possible positive correlation between the encoding proteins of high molecular HSPs and chalky endosperm.

As to the possible mechanisms explaining the high content of chalky grains in 2010, it is worth to note that the levels of some glutathione redox isoforms in oCG 2010 were, all of them, higher than oCG 2009 (Fig. 3c). Glutathione, implicated in the antioxidant defense through the ascorbate/GSH cycle, plays a key role in maintaining the homeostasis of reactive oxygen species (ROS). Glutathione S-transferases (GSTs) are ubiquitous enzymes encoded by a large family of genes, which play an important role in cellular detoxification to a wide variety of endobiotic and xenobiotic substrates by conjugating the tripeptide glutathione. GSTs have been found to be differentially regulated by dehydration (Bianchi et al. 2002). However, antioxidant systems cannot completely prevent the deleterious effects of ROS. In this study, nine of the identified proteins are implicated in redox homeostasis-related functions in chalky grains, including glutathione transferase (GT), GSTs and glutathione peroxidase (GPX). Therefore, this would indicate that the ROS scavenging system may be activated in rice chalky kernels to alleviate such oxidative damage and to enhance high-temperature tolerance, mostly in 2010. Two proteins, GPX and GST, which are involved in the glutathione-ascorbate cycle for removing H_2_O_2_, showed the same expression patterns (Figs. 3c). GST and GPX can reduce H_2_O_2_ to its corresponding hydroxyl compounds to remediate oxidative membrane damage, and their expression showed the increases in abundance in chalky tissues. Some of these proteins were consistent with those observed in the rice (Lin et al. 2014) and maize (Luo et al. 2010) in response to stress in developing kernel tissues. Thus, our results, in conjunction with those previously reported (Liu et al. 2010; 2011; Lin et al. 2014), show that the up-regulation of these proteins, more pronounced in high-temperature conditions, suggest a close relation between redox homeostasis and the enhancement of chalky grains frequency. Recent investigation revealed that a heat-tolerant cultivar of rice exhibits a characteristic high expression of superoxide dismutase MSD1. In addition, the grain quality of transgenic overexpressor plants with the maize *Ubiquitin-1* promoter fused to *MSD1* was significantly improved in comparison with the wild type under heat stress after heading (Shiraya et al. 2015). We infer that the timely enhancement of H_2_O_2_ level by MSD1 under high-temperature stress is probably important, which acts as a signal that rapidly can promote the expression of stress-response proteins (Shiraya et al. 2015; Mitsui et al. 2016).

Among various additional chalky grain stress-related proteins, the expression of prohibitin and DREPP2 proteins were most remarkably increased under a high temperature -oCG 2010- (Fig. 3d). Moreover, a characteristic behavior of expression of prohibitin which may have multiple functions including mitochondrial chaperone activity (Tatsuta et al. 2005; Van Aken et al. 2009) was up-regulated in oCG 2010, while it was down-regulated under oCG 2009. To our knowledge, this study is the first to provide insights into the differential expression of prohibitin proteins in opaque chalky part collected from rice grains following heat stress season. Prohibitin have been shown to play central roles in cell cycle regulation, receptor-mediated signaling at the cell surface, aging, apoptosis, and plant development and senescence (Berger and Yaffe 1998; Coates et al. 1997; McClung et al. 1992, 1995; Nijtmans et al. 2002; Piper et al. 2002; Chen et al. 2005). Recent studies using muscovy ducks suggested changes in the abundance of the mitochondrial protein chaperone, prohibitin, into thermal tolerance related to energy metabolism (Zeng et al. 2013).

**Fig. 4:**
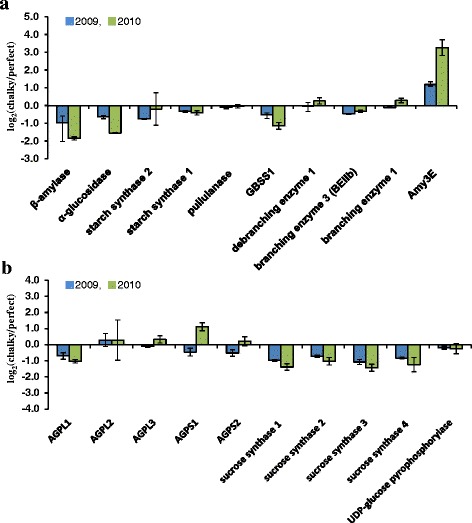
Proteome of starch metabolism in chalky grains. Center part of perfect grains and opaque part of chalky grains harvested in 2009 and 2010 were subjected to protein extraction, followed by in solution shotgun proteomic analysis with iTRAQ labeling. **a** starch synthesis and degradation; **b** ADP-glucose supply. Values are represented as mean ± s.d. (*n* = 3)

The biosynthesis of starch is the major determinant of yield in cereal grains (Emes et al. 2003). Furthermore, the main constituent of the rice grain is starch, thus the grain filling capacity is determined mainly by the starch-synthesizing capability of endosperm. Proteins associated with carbohydrate metabolism, especially starch synthesis, showed altered expression patterns induced by high temperature. Among them, the α-amylase isoform, Amy3E (AmyII-3), was dramatically up-regulated in oCG 2010 (Fig. 4a). As expected, the expression level of granule-bound starch synthase 1 (GBSS1), starch branching enzyme (SBE) BEIIb, soluble starch synthases (SS), and sucrose synthases (SuSy) were down-regulated in the chalky grains (Fig. 4a, b). It has been observed that the expression of starch-synthesis-related genes repressed under high-temperature conditions (Yamakawa et al. 2007). In the growing rice grains, the concerted functioning of multiple forms of granule-bound (GBSS) and SS, SBE and debranching enzymes (DBE), together with the ADPG supplying strength, determine the overall grain-filling capacity (Su 2000). Thus, the impaired balance of starch biosynthesis and degradation at oCG 2010 may increase the rate of grains to appear chalky because of the imperfect filling of endosperm cells with starch granules.

Noteworthy, high-temperature stress can lead to changes in the other proteins involved in lipid (Fig. 2b, Additional file 1: Table S1) and glycolysis metabolic processes (Fig. 2b), suggesting that primary metabolism might have been inhibited in chalky grains. Indeed, we found the lipid metabolism-related proteins, caleosin and sterol carrier protein, were differentially expressed in oCG, which may aid the mechanisms of chalkiness formation. The down-regulation of these proteins might retard the metabolic speed of fatty acid synthesis, under high-temperature conditions, in chalky grains. Because caleosin is associated with the endoplasmic reticulum and/or oil bodies (OBs) in seed embryo development (Næsted et al. 2000), promotes specific interaction of OBs with vacuoles and facilitates access to triacylglycerides, to serve as energy source, by lipases (Poxleitner et al. 2006). Likewise, sterol carrier protein, peroxisomal lipid protein, might affect the transfer of lipids between membranes, β-oxidation and glyoxylate pathways, and hence the normal development and morphology of grains (Zheng et al. 2008). Thus, down-regulation of lipid metabolism related proteins probably contribute to the chalkiness formation in grains of 2009-to-2010. Further detailed investigations, including lipid profiling may provide us with additional clues to the biological function of those proteins in chalkiness formation.

### Glycomic characteristics of chalky grains caused under different temperature conditions

**Fig. 5:**
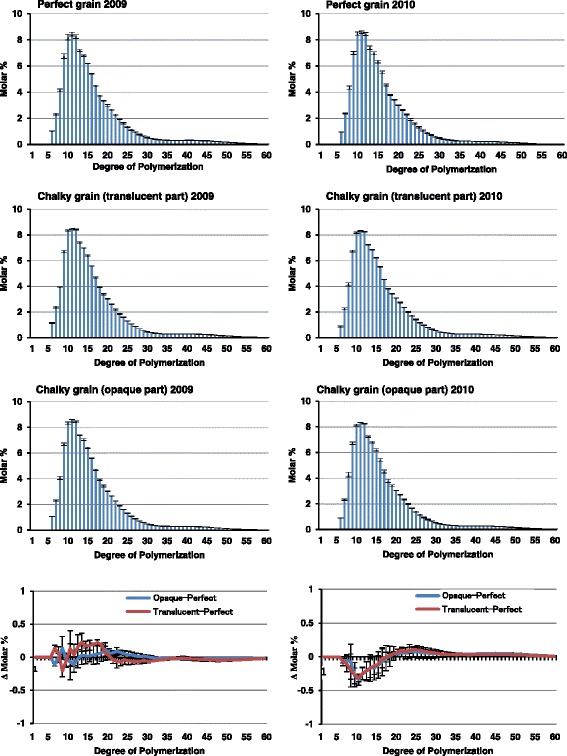
Chain-length distributions of perfect and chalky grain amylopectins of rice. Rice grains were harvested in 2009 and 2010 that the average temperatures of grain filling periods were 24.4 and 28.0 °C, respectively. Center part of perfect grains and translucent and opaque parts of chalky grains were subjected to starch extraction, followed by APTS labeling and capillary electrophoretic analysis. Bottom panels show differences in the chain distributions of amylopectins in perfect and chalky grains. Blue lines, differences between the opaque part of chalky grain and perfect grain; red lines, differences between the translucent part of chalky grain and perfect grain. Values are represented as mean ± s.d. (*n* = 3)

Starch quality is an important parameter in determining the quality of rice grains. Numerous investigations have revealed that the environmental temperature at the grain filling stage apparently alters the starch composition in rice grains (Mitsui et al. 2016). Furthermore, the content and fine structure of amylose and amylopectin in starch affect the physicochemical characteristics (such as viscosity) and texture properties of the rice grains (Muench et al. 1997). We have previously determined amylose contents and chain-length distributions of starches prepared from the translucent and opaque parts of perfect and chalky grains of Koshihikari harvested in 2009 and 2010 using a fluorescence labeling followed by HPLC size-exclusion chromatography. These data have suggested that the contents of amylose chain-length distributions between the translucent and opaque parts of rice grains in both seasons were similar. Although, slight differences in the contents of amylose and long B chains of amylopectin were observed in 2009 and 2010 (Tsutsui et al. 2013). To confirm the results, we conducted a further analysis of chain-length distribution of amylopectin of PG 2009, tCG 2009, oCG 2009, PG 2010, tCG 2010, and oCG 2010 using the fluorescence capillary electrophoresis (FCEP) method. As shown in Fig. 5, these analyzes revealed clearly that the differences in the chain-length distribution of starch among the perfect grains and the translucent and opaque parts of chalky grains were below the detection limit. In oCG and tCG 2010, the relative amounts of amylopectin middle-size chains (DP20-30) slightly increased while short-chain DP5-15 decreased, although no differences between two parts in the same chalky grains were observed (Fig. 5). Therefore, the reason for the appearance of loosely-packed round-shaped starch granules of the chalky grains must be attributed to factors other than chain-length distribution of amylopectin.

High-temperature stress decreased the amylose contents and the weight ratio of A+ short B chains to long B chains of amylopectin (Asaoka et al. 1984; Inouchi et al. 2000). In line with these conclusions, previous results of our group obtained by analyzing the starch composition of Koshihikari grains harvested in moderate and high-temperature conditions were not contradictory to the conclusions mentioned above (Tsutsui et al. 2013). Furthermore, transcriptomic analysis of developing rice seeds has demonstrated that the expression of several starch synthesis-related genes including *GBSSI*, *BEIIb*, ADP-glucose pyrophosphorylase (*AGPS2b, AGPS1 and AGPL2*) and ADP-glucose translocator (*BT1-2*) were decreased under high-temperature condition (Yamakawa et al. 2007). It is well known that the *amylose-extender* (*ae*) mutant of rice, that is deficient in *BEIIb* gene, exhibited a severe chalky phenotype of grain. The *ae* mutant revealed that the mutation in the gene for BEIIb specifically altered the structure of amylopectin in the endosperm by reducing short chains with degree of polymerization of 17 or less, with the greatest decrease in chains with degree of polymerization of 8 to 12 and enriched in long chains with DP more than 19 (Nishi et al. 2001). The chalkiness of *ae* mutant was alleviated by manipulation of BEIIb activity (Tanaka et al. 2004; Abe et al. 2014), suggesting that the unusual expression of *BEIIb* could be one of the key factors causing the grain chalkiness. However, Yamakawa et al. (2007) showed that the reduction of amylase content and amylopectin side chain by high temperature was not correlated to the grain chalkiness in rice cultivars ranging from high-temperature tolerance to high temperature sensitive. Thus, the relevance of the starch fine structure to the chalkiness of grain under high-temperature stress is obscure.

**Fig. 6:**
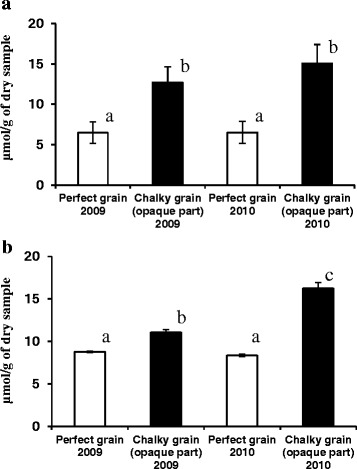
Soluble starch with glucose (**a**) and sucrose (**b**) contents in the perfect and chalky (opaque part) grains harvested in 2009 and 2010. Values are represented as mean ± s.d. (*n* = 6–12). Histograms with the same letter do not differ significantly (p ≤0.05)

The function of starch synthesis-degradation in stem seems to serve as an important regulatory role of rice grain filling. The starch synthesis in grains starts from sucrose translocated from leaf cells. The contents of soluble starch in the opaque parts (oCG 2009 and oCG 2010) were remarkably high in comparison with the corresponding translucent parts of perfect grains (PG 2009 and PG 2010) (Fig. 6a), indicating that amylolytic enzyme exists and works in the opaque parts of chalky grains. Soluble sugar contents including sucrose were significantly increased in oCG 2010 (Fig. 6b). The sucrose contents of oCG under high temperature were higher than PG and those in 2009. It is thus likely that the downregulation of SuSy proteins under heat stress is one reason for the accumulation of sucrose in chalky grains. Another striking alteration involving downregulation of the sucrose transporter (*OsSUT1*) under heat stress favored the accumulation of sucrose in these organs (Phan et al. 2013). Another possible reason explaining the accumulation of sucrose is to alleviate water stress as a consequence of osmotic adjustment. Recent findings during rice grain filling, under simultaneous occurrence of high temperature and water deficit, showed that sugars to be synthesized to starch kept accumulating in vacuoles and cytosol in the cells (Wada et al. 2014), so that the cells could be expanded by maintaining water volume in size and cell Ψp (Morgan 1977; Meyer and Boyer 1981), followed by starch accumulation.

**Fig. 7:**
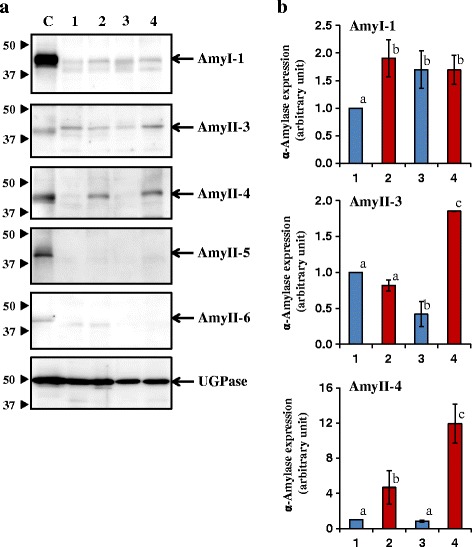
Changes in the expression of α-amylase isoforms in central opaque area of chalky grains harvested in 2009 and 2010. **a** Immunoblotting images. Center part of perfect grain and opaque part of chalky grain were subjected to protein, followed by SDS-PAGE and immunoblotting with specific antibodies. C, germinating seeds after 5 days of imbibition; lane 1, perfect grain in 2009; lane 2, chalky grain in 2009; lane 3, perfect grain in 2010; lane 4, chalky grain in 2010. **b** Quantitation of α-amylase isoforms expression. Amount of each α-amylase isoform in perfect grains was normalized to 1 unit. The perfect proteins of AmyII-5 and AmyII-6 were not detected. Values are represented as mean ± s.d. (*n* = 3). Histograms with the same letter do not differ significantly (p ≤0.05)

We further determined the amounts of α-amylase isoforms in PG 2009, oCG 2009, PG 2010, and oCG 2010 by Western blotting (Fig. 7a, b). The results showed that AmyII-3 (Amy3E) and AmyII-4 (Amy3D) were highly expressed in oCG 2010 compared with PG 2010, and AmyI-1 (Amy1A) and AmyII-4 were significantly expressed in oCG 2009 compared with PG 2009 (Fig. 7b). It is noteworthy that in developing seeds of rice, the expression of α-amylase genes *Amy1A*, *Amy1C*, *Amy3D* and *Amy3E* were induced under high-temperature stress and its suppression, through RNA interference (RNAi) strategy, ameliorated rice grain damage such as chalkiness (Hakata et al. 2012). Asatsuma et al. (2005) reported that ectopic overexpression of α-amylases such as Amy1A and Amy3D produced chalky grains even under ambient temperature. As the extent of the decrease in chalky grains was highly correlated to decreases in the expression of Amy1A, Amy1C, Amy3A and Amy3B. Furthermore, studies have demonstrated that AmyI-1 and AmyII-4 proteins existed in the outer layers (100 to 80 % fractions) of rice grain (cv. Koshihikari), while α-glucosidase and AmyII-3 were mainly detected in the inner layers (80 to 0 % fractions) by immunoblotting with the specific antibodies. Likewise, starch-component profiles are impacted by changes in air temperature. The overall experimental results revealed that the degradation of starch accumulating in the developing grains by amylase under high temperature is an another layer of regulation causing the chalkiness.

## Conclusions

In this study, the higher coverage of rice grains proteome by the iTRAQ/Shotgun strategy offered a good opportunity to discover more stress-related proteins involved in chalkiness tissue. Under field conditions, the differentially expressed proteins in opaque chalky grains under moderate (2009) and heat stress (2010) suggest three highly enriched functional terms, i.e. LEA proteins, HSPs, and glutathione peroxidase and S-transferase. These analyzes give us a better understanding about the chalky dynamics with global-mean warming of roughly 4 °C in rice grains. Furthermore, the formation of chalky grain under high-temperature season is also triggered by alterations in proteins associated with carbohydrate metabolism and starch structure. Thus, down-regulation of genes involved in starch biosynthesis and the involvement of α-amylase isoforms in the central opaque area of chalky grains were elucidated under elevated temperature. Our results provide new insights into proteome and glycome characterization in perfect and chalky grain in high-temperature scenarios at the field. The proteins identified here provide a basis to elucidate further the molecular mechanisms underlying the chalkiness under elevated temperature and may reveal useful targets in climate change scenarios studies and strategies.

## Methods

### Plant materials

Rice grains (*Oryza sativa* L. cv. Koshihikari) grown at Sanjo city (Niigata, Japan) were harvested in two consecutive years, 2009 and 2010. The average temperatures during the heading and ripening period of Koshihikari in the area of 2009 and 2010 were 24.4 and 28.0 °C, respectively. Grain quality (chalky or perfect) was determined with a rice grain grader (RGQI20A, Satake, Hiroshima, Japan). “Perfect” grains (PG) that exhibited a transparency and “chalky” grains (tCG) that contain opaque part(s) (oCG) within the endosperm were selected from these harvested grains on a viewer (Fujicolor light box New-5,000 Inverter, Fuji film Co., Tokyo, Japan). All the samples were stored in a temperature range of 4 to 10 °C until the experiment was carried out.

### Scanning electron microscopy (SEM)

Brown rice grain was cracked with a razor blade, and the cracked surface was coated with gold for 90 s using a vaporizer (IB-3, EIKO, Tokyo, Japan) and subjected to a Scanning Electron Microscope (JSM6510LA, JEOL, Tokyo, Japan). Observation conditions were as follows: acceleration voltage, 10 kV; magnification, 1,000-10,000. SEM analysis was run using three biological replicates with at least four technical repetitions of developing grains per replication of the mounted specimens.

### Proteomic analysis

Two hundred mg of starchy endosperm prepared from the opaque part of chalky grains or the corresponding part of perfect grains were ground in liquid nitrogen to fine powder and then suspended in 7 M urea, 2 M thiourea, 3 % (w/v) CHAPS, 1 % (v/v) Triton X-100, and 10 mM DTT. The suspensions were centrifuged at 10,000× *g* at 4 °C for 5 min. The supernatants were mixed with 1/10 volume of 100 % (w/v) trichloroacetic acid, incubated on ice for 15 min, and centrifuged at 10,000× *g* at 4 °C for 15 min. The resulting protein precipitates were washed 3 times in ice-cold acetone and resuspended in 8 M urea. Protein concentration was determined by the Pierce 660 nm Protein Assay Kit (Thermo Fisher Scientific) using bovine serum albumin (BSA) as a standard.

The procedure of quantitative shotgun proteomic analysis was essentially identical as described previously (Shiraya et al. 2015). Each protein preparation (50 μg) was digested in 20 μl of endoproteinase Lys-C (1 μg μl^−1^) at 37 °C for 3 h, then diluted to 10 times volume by ultrapure water (18.2 MΩ cm). The diluted samples were further digested in 200 μl of trypsin (1 μg μl^−1^) at 37 °C for 12 h. iTRAQ labeling of peptides were carried out with 4-plex iTRAQ tags the manufacturer's protocol (Sciex), and the resultant 4 iTRAQ-labeled peptide samples were mixed. iTRAQ analysis was performed by using a DiNa-A-LTQ-Orbitrap-XL system. The iTRAQ labeled peptides were loaded on a trap column (HiQ sil C-18 W-3; 0.5 mm i.d. × 1 mm, 3 μm particle size) with buffer A consisting of 0.1 % (v/v) formic acid and 2 % (v/v) acetonitrile in water using a DiNa-A system (KYA Tech., Tokyo, Japan). A linear gradient from 0 to 33 % buffer B (0.1 % formic acid and 80 % acetonitrile in water) for 600 min, 33 to 100 % B for 10 min and back to 0 % B in 15 min was applied, and peptides eluted from the HiQ sil C-18 W-3 column were directly loaded on a separation column (MonoCap C18 High Resolution 2000; 0.1 mm i.d. x 2000 mm, 2 μm pore size). The separated peptides were introduced into an LTQ-Orbitrap XL mass spectrometer (Thermo Fisher Scientific) with a flow rate of 300 nL min^−1^ and an ionization voltage 1.7–2.5 kV.

Liquid chromatography-MS/MS (LC-MS/MS) spectrometer was operated using Xcalibur 2.0 software (Thermo Fisher Scientific). The mass range selected for MS scan was set to 350–1,600 m/z and the top three peaks were subjected to MS/MS analysis. Full MS scan was detected in the Orbitrap, and the MS/MS scans were detected in the linear ion trap and Orbitrap. The normalized collision energy for MS/MS was set to 35 eV for collision-induced dissociation (CID) and 45 eV for higher-energy C-trap dissociation (HCD). High resolution of Fourier transform mass spectrometer (FTMS) was maintained at 60,000 resolution. Divalent or trivalent ions were subjected to MS/MS analysis in dynamic exclusion mode, and proteins were identified with Proteome Discoverer v. 1.4 software and the SEQUEST HT (Thermo Fisher Scientific) and MsAmanda (Dorfer et al. 2014) search tool using the UniProt (http://www.uniprot.org/) *O. sativa* subsp. *japonica* database (63,535 proteins) with the following parameters: enzyme, trypsin; maximum missed cleavages site, 2; peptide charge, 2+ or 3+; MS tolerance, 5 ppm; MS/MS tolerance, ±0.5 Da; dynamic modification, carboxymethylation (C), oxidation (H, M, W), iTRAQ 4-plex (K, Y, N-terminus). False discovery rates were <1 %.

### Western blotting

The starchy endosperm samples (500 mg) were powdered in liquid nitrogen and suspended in 1 mL of 10 mM Tris–HCl (pH 7.5), 1.0 mM CaCl_2_ and 0.1 % (w/v) Triton X-100 for 15 h at 4 °C. The suspension was centrifuged at 15 000× *g* for 10 min at 4 °C. An aliquot of the supernatant was subjected to sodium dodecyl sulfate - polyacrylamide gel electrophoresis (SDS-PAGE) in 12 % separation gels. After electrophoresis, the separated proteins were transferred to polyvinylidene fluoride membranes (Hybond-P; GE Healthcare, USA). The membranes were incubated in 15 mM phosphate-buffered saline (pH 6.8) containing 0.1 % Tween-20 and 5 % skim milk for blocking, and then reacted with specific antibodies: anti-rice α-amylase isoform AmyI-1 (rabbit serum; 1:10,000), AmyII-3 (mouse serum; 1:500), AmyII-4 (rabbit serum; 1:5,000) (Itayagoshi et al. 2015), AmyII-5, AmyII-6 (rabbit serum; 1:5000) (Nanjo et al. 2004) and UDPglucose pyrophosphorylase (Nanjo et al. 2004). Horseradish peroxidase-conjugated anti-rabbit IgG (Nacalai Tesque, Japan) and anti-mouse IgG (MP Biomedicals, USA) were used as secondary antibodies. The α-amylase immunobloting assay is isoforms specific, as there is no cross-reactivity of the test among isoforms AmyI-1, Amy II-4 (Mitsui et al. 1996) and Amy II-3 (Tsuyukubo et al. 2012), due to antibody specificity used in the test. The immunoreactive bands were visualized using chemiluminescence reagent (Amersham, UK), and quantified by LAS-3000 molecular imager (Fujifilm, Japan) (Asatsuma et al. 2005).

### Measurement of chain-length distribution of starch

The method determining chain-length distribution of starch was essentially identical to the procedure described by Fujita et al. (2012). Starchy endosperm (5 mg) was ground with a mortar and pestle and the powder was boiled in 1.5 ml of methanol for 10 min. The suspension was centrifuged at 3,000 x *g* and room temperature for 5 min. The pellet was re-suspended in 1.5 ml of 90 % (v/v) methanol, and centrifuged at 3,000 x *g* for 5 min at room temperature. After removing methanol, the pellet was suspended with 143 μl of ultrapure water, and mixed with 7.5 μl of 5 N NaOH and boiled for 5 min. The α-glucan sample was desalted and subjected to hydrolysis with isoamylase (0.03 U/mg of amylopectin in 40 mM acetate buffer, pH 4.4) at 37 °C for 12 h. The chain length distributions of α-glucans from endosperm were analyzed using the fluorescence capillary electrophoresis (FCEP) method of O´Shea and Morell (1996) in a P/ACE MDQ Carbohydrate System (Beckman Coulters, CA, USA).

### Soluble starch and sucrose contents

Briefly, 50 mg of starchy endosperm was treated with 2 ml of 80 % ethanol in boiling water bath for 1 min, and the mixture was centrifuged at 10,000x *g* for 10 min. The ethanol extraction was carried out three times. The ethanol fraction containing soluble glycan and free sugars was dried-up and then subjected to sugar assays. The soluble glycan was hydrolyzed by 5 unit of amyloglucosidase and 1 unit of α-amylase, and the released glucose from soluble glycan was measured by a coupled enzyme reaction using hexokinase (HK) and Glc-6-P dehydrogenase (G6PDH) (Guglielminetti et al. 1995). The assay mixture, composed of 100 mM Tris–HCl (pH 7.6), 3 mM MgCl_2_, 2 mM ATP, 0.6 mM NAD^+^, 1 unit of HK and 1 unit of G6PDH, was incubated at 37 °C for 30 min. After cooling, absorbance was measured at 340 nm using a spectrophotometer (Hitachi U-2900). Sucrose was first broken down using 85 units invertase (in 15 mM sodium acetate, pH 4.6) and the resulting glucose and fructose were assayed as described above.

## Abbreviation

ADPG, adenosine diphosphate D-glucose; AGP, ADPG pyrophosphorylase; AGPP, ADPG pyrophosphatase; Amy, amylase; BE, branching enzyme; DBE, debranching enzyme; G6PDH, Glc-6-P dehydrogenase; GBSS, granule bound starch synthase; GPX, glutathione peroxidase; GST, glutathione S-transferases; GT, glutathione transferase; HK, hexokinase; HSPs, heat shock proteins; LEA, late embryogenesis abundant; oCG, opaque chalky grain; PG, perfect grain; PPase, pyrophosphorylase; ROS, reactive oxygen species; SDS, sodium dodecyl sulfate; sHSPs, small HSPs; SPS, sucrose phosphate synthase; SS, starch synthase; SuSy, sucrose synthase; tCG, transparent chalky grain; UDPG, UDPglucose; UGPase, UDP-glucose pyrophosphorylase.

## Additional file

Additional file 1: Table S1. Proteomic comparison of chalky and perfect grains harvested in 2009 and 2010. s.d., standard deviation; # AAs, the number of amino acids; MW, the calculated molecular weight; pI, the calculated isoelectric point; ΣCoverage, the percentage of sequence coverage. (XLSX 62343 kb)
